# Activated platelets release sphingosine 1-phosphate and induce hypersensitivity to noxious heat stimuli *in vivo*

**DOI:** 10.3389/fnins.2015.00140

**Published:** 2015-04-22

**Authors:** Daniela Weth, Camilla Benetti, Caroline Rauch, Gerhard Gstraunthaler, Helmut Schmidt, Gerd Geisslinger, Roger Sabbadini, Richard L. Proia, Michaela Kress

**Affiliations:** ^1^Division of Physiology, Department of Physiology and Medical Physics, Medical University of InnsbruckInnsbruck, Austria; ^2^Pharmazentrum Frankfurt/ZAFES, Institute of Clinical PharmacologyFrankfurt, Germany; ^3^Lpath, Inc.San Diego, CA, USA; ^4^Genetics of Development and Disease Branch, National Institute of Diabetes and Digestive and Kidney DiseasesBethesda, MD, USA

**Keywords:** platelet, post-operative pain, sphingosine 1-phosphate, heat nociception, S1P receptors

## Abstract

At the site of injury activated platelets release various mediators, one of which is sphingosine 1-phosphate (S1P). It was the aim of this study to explore whether activated human platelets had a pronociceptive effect in an *in vivo* mouse model and whether this effect was based on the release of S1P and subsequent activation of neuronal S1P receptors 1 or 3. Human platelets were prepared in different concentrations (10^5^/μl, 10^6^/μl, 10^7^/μl) and assessed in mice with different genetic backgrounds (WT, S1P_1_^fl/fl^, SNS-S1P_1_^−/−^, S1P_3_^−/−^). Intracutaneous injections of activated human platelets induced a significant, dose-dependent hypersensitivity to noxious thermal stimulation. The degree of heat hypersensitivity correlated with the platelet concentration as well as the platelet S1P content and the amount of S1P released upon platelet activation as measured with LC MS/MS. Despite the significant correlations between S1P and platelet count, no difference in paw withdrawal latency (PWL) was observed in mice with a global null mutation of the S1P_3_ receptor or a conditional deletion of the S1P_1_ receptor in nociceptive primary afferents. Furthermore, neutralization of S1P with a selective anti-S1P antibody did not abolish platelet induced heat hypersensitivity. Our results suggest that activated platelets release S1P and induce heat hypersensitivity *in vivo*. However, the platelet induced heat hypersensitivity was caused by mediators other than S1P.

## Introduction

Acute tissue injury, e.g., occurring as a consequence of local trauma or surgery, usually initiates local platelet aggregation, blood coagulation, closure of blood vessels, and later the classical symptoms of inflammation, that is redness, swelling, local warming, loss of function, and pain. Post-injury pain results from an excitation and sensitization of nociceptor primary afferent nerve fibers by inflammatory mediators accumulating at the site of inflammation. Such mediators are released by activated immune cells, including mast cells, neutrophils and macrophages, but also by platelets, which are attracted and activated by cell-attached or diffusible components of the traumatized tissue (Basbaum et al., 2009).

Platelets are among the first defense line to arrive at the site of injury and in addition to their function in hemostasis they play important roles during inflammatory processes (Semple et al., 2011). Numerous mediators are released after platelet activation including adenosine 5′-diphosphate (ADP), adenosine 5′-triphosphate (ATP), histamine, serotonin (5-hydroxytryptamine, 5-HT), thromboxane A2 (TXA2), platelet activating factor (PAF), interleukin-1β (IL-1β) and many of these compounds activate nociceptors and induce pain and hyperalgesia (for review see Petho and Reeh, 2012). Increasing evidence is available for the rapid release of sphingosine 1-phosphate (S1P) (Yatomi et al., 2000), an ubiquitously expressed bioactive lipid mediator, which is generated intracellularly from sphingosine via phosphorylation by sphingosine kinases 1 or 2 (Spiegel and Milstien, 2003). S1P is abundantly stored in platelets, as these cells do not express enzymes for S1P degradation (Spiegel and Milstien, 2011). Upon activation platelets release S1P (Yatomi et al., 1995; Ulrych et al., 2011), which may then bind to one of its cognate G protein-coupled receptors (S1P_1−5_) located in the plasma membrane of its target cells (Spiegel and Milstien, 2011). In recent studies S1P has been demonstrated to induce nociceptor sensitization and excitation and these effects are transduced by S1P_1_ and S1P_3_ receptors, respectively (Zhang et al., 2006; Chi and Nicol, 2010; Mair et al., 2011; Camprubí-Robles et al., 2013).

In the present study, we assessed a possible S1P mediated pronociceptive action of activated human platelets in an *in vivo* mouse model. For this purpose, we intracutaneously injected activated human platelet preparations into the mouse hind paw and monitored reflex withdrawal responses to standard heat stimuli at different time points post-injection. Platelet injection induced significant hypersensitivity to heat stimuli, which persisted for several hours. Furthermore, we quantified the S1P content of platelet preparations and investigated a possible correlation of the platelet concentration with the degree of hypersensitivity. Finally, we assessed whether the observed increase in heat sensitivity was based on a direct activation of nociceptors via S1P_1_ or S1P_3_ receptors in two different transgenic mouse strains, either lacking S1P_1_ in Nav1.8 expressing nociceptive neurons (SNS-S1P_1_^−/−^) or with a global null mutation for S1P_3_, and whether neutralization of S1P in the platelet preparations, as published previously in a different model (Finley et al., 2013), prevented the development of heat hypersensitivity upon platelet injection.

## Materials and methods

### Ethics statement

All animal experiments were performed in accordance with national Austrian law and ethical guidelines of the IASP (International Association for the Study of Pain) (Zimmermann, 1986), and with permission of the Austrian Bundesministerium für Wissenschaft und Forschung (BMWF) (BMWF-66.011/0051-II/10b2008; BMWF-66.011/0113-II/3b/2010; GZ 66.011/85-C/GT/2007).

### Genetically modified mice

All experiments were performed in age-matched awake, unrestrained, male C57BL/6J mice (8–12 weeks old), which were housed on a 12 h light/dark cycle with free access to food and water. Wt, S1P_1_^fl/fl^, SNS-S1P_1_^−/−^ and S1P_3_^−/−^ mice were used for the experiments.

Mice homozygous for the lox P flanked exon 2 allele of the S1P_1_ receptor gene (S1P_1_^fl/fl^) and global S1P_3_ receptor null mutant mice (S1P_3_^−/−^) have been described previously (Ishii, 2001; Allende et al., 2003; Kono et al., 2004). Since mice with a global null mutation of S1P_1_ are not viable, S1P_1_^fl/fl^ mice were cross-bred with SNS-Cre mice (Agarwal et al., 2004) to obtain SNS-Cre:S1P_1_^fl/fl^ mice (subsequently referred to as SNS-S1P_1_^−/−^) which selectively lack the S1P_1_ receptor in Nav1.8 channel-expressing nociceptive neurons (Mair et al., 2011).

### Platelet preparation

Human platelet concentrates were a generous gift of the Blood Bank of the University Medical Center, Innsbruck. Individual bags held at least 2 × 10^11^ platelets per bag. The content was centrifuged (20 min at 10.6 g and 10°C) and the supernatant was removed. Subsequently, platelets were resuspended in 20 ml PBS (Dulbecco's PBS w/o Ca^2+^ and Mg^2+^; PAA, Pasching, Austria). Aliquots of different concentrations (10^5^, 10^6^ and 10^7^ platelets/μl) were prepared and frozen at −80°C. Prior to injection aliquots were defrosted and through this procedure platelets were activated (Rauch et al., 2011). In addition thawed platelets were sonicated (20 min) and centrifuged (20 min at 10.6 g and 10°C) to obtain supernatant which was immediately used for further experiments. The murine monoclonal anti-S1P antibody, Sphingomab/LT1002 and its inactive isotype mAb control, LT1017, (Lpath Incorporated, San Diego, CA, USA, O'Brien et al., 2009) were added to the activated platelet preparations (10^7^ platelets/μl) immediately prior to injection with a final concentration of 0.03 μg antibody per 1 μl of platelet preparation as published previously (Finley et al., 2013).

### Heat sensitivity

Heat sensitivity was assessed by using the Hargreaves test (Hargreaves et al., 1988). A radiant heat source delivering an increasing heat stimulus was focused under the plantar surface of the hindpaw. The time until the mouse responded to stimulation with reflex paw withdrawal (paw withdrawal latency [PWL] in seconds) was measured automatically with an algesiometer (Ugo Basile, Comerio, Italy). Each hindpaw was tested at least three times and the mean withdrawal latency was calculated. The time interval between two trials on the same hindpaw was at least 1 min. Baseline measurements were taken the day before and on the day of injection. Platelets, platelets + LT1002, platelets + LT1017 or PBS were injected intracutaneously in a total volume of 10 μl using a Hamilton syringe. Changes in heat sensitivity were assessed 30 min, 1, 2, 3, and 24 h post-injection. Experimenters were blinded either to the genotype of the mice or to the injected substance.

### S1P concentration analysis

Sphingolipids were liquid-liquid extracted from a 50 μL sample using methanol:chloroform:HCl (15:83:2, v/v/v). After evaporation of the organic layer and reconstitution in methanol, sample analysis was performed by using liquid chromatography–electrospray ionization-tandem mass spectrometry (LC–ESI-MS/MS). For HPLC an Agilent 1100 Series binary pump and degasser connected to an HTC PAL autosampler (Chromtech, Idstein, Germany) was used. A triple quadrupole mass spectrometer 4000 Q TRAP equipped with a Turbo V source ion spray operating in positive ESI mode was used for detection (AB Sciex, Darmstadt, Germany). High purity nitrogen for the mass spectrometer was produced by the nitrogen generator NGM 22-LC/MS (cmc Instruments, Eschborn, Germany).

Chromatographic separations were obtained under gradient conditions using a Luna C18 column (150 × 2 mm i.d., 5 μm particle size) with a same material guard column (4 mmL × 2 mm i.d.) (Phenomenex, Aschaffenburg, Germany) and eluent A (water/formic acid (100:0.1 v/v)) and eluent B [acetonitrile/tetrahydrofuran/formic acid (50:50:0.1 v/v)]. The flow rate was set at 0.3 ml/min and the runtime at 14 min.

The mass spectrometer was operated in the positive ionization mode with an electrospray voltage of 5400V at 400°C. Precursor-to-product ion transitions of m/z 380.1→264.2 for S1P (collision energy 23 V), m/z 300.3→252.3 for SPH (26 V), m/z 302.2→60.1 for SAPH (43 V), m/z 382.1→284.1 for SA1P (20 V), m/z 366.2→250.1 for C17-S1P (internal standard, 23 V) and m/z 286.2→238.1 for C17-SPH (internal standard, 26 V) were used as quantifier for the multiple reaction monitoring (MRM) with a dwell time of 100 ms. All quadrupoles were working at unit resolution. Quantification was performed with Analyst Software V1.4 (AB Sciex, Darmstadt, Germany) using the standard-addition method.

### Statistical analysis

For detailed statistical analysis the SigmaStat3 software package was used. Data are presented as mean ± s.e.m. Two-way repeated measures ANOVA followed by Tukey *post-hoc* test or Bonferroni *post-hoc* test and Mann-Whitney *U*-test for comparisons between groups were used. Differences were considered statistically significant at *p* < 0.05.

## Results

### Platelet induced sensitivity to heat stimuli and S1P content of platelet preparations

**Figure 1:**
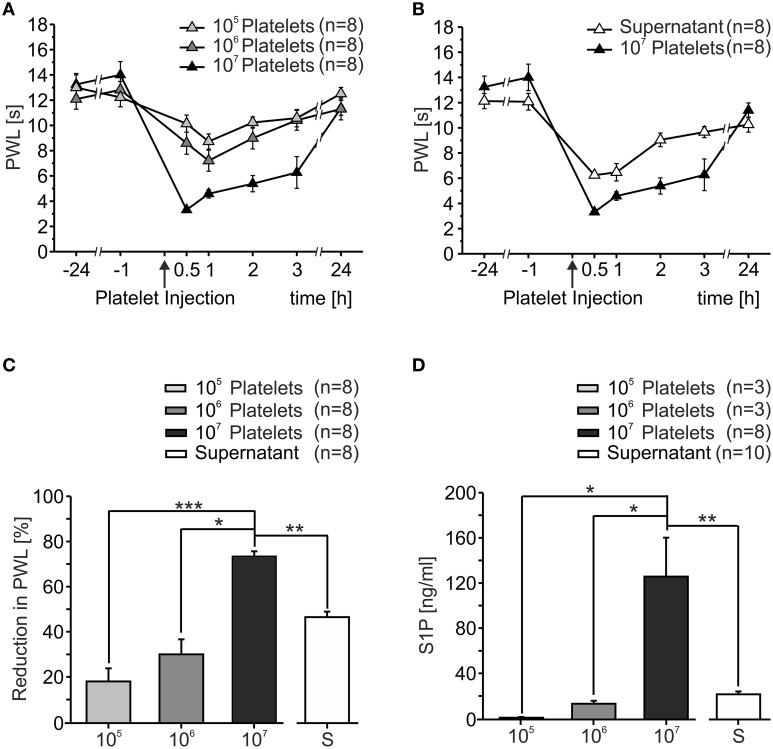
**Activated platelets increased thermal sensitivity *in vivo.* (A)** After intracutaneous injection of platelet preparations (10 μl) in different concentrations a dose-dependent reduction of paw withdrawal latency (PWL) was observed. Heat sensitization recovered to baseline after 24 h. **(B)** Injection of the supernatant of sonicated platelets led to a reduction in PWL as well. **(C)** Quantification of PWL shown in **(A,B)** 1 h after injection (^*^p < 0.05, ^**^p < 0.01, ^***^p < 0.001; Mann Whitney *U*-test). **(D)** S1P concentration in platelet preparations as measured with HPLC. S1P concentration was directly proportional to platelet concentration (^*^p < 0.05, ^**^p < 0.01; Mann Whitney *U*-test).

In order to assess the effect of activated platelets on heat sensitivity *in vivo*, platelet preparations of different concentrations (10^5^/μl, 10^6^/μl, 10^7^/μl) were injected into the hindpaw of C57BL/6J mice. Upon injection, mice displayed little to no nocifensive behavior (data not shown). Sensitivity to heat stimuli before and after platelet injection was measured as PWL and a dose-dependent heat hypersensitivity after platelet injection was observed (Figure 1A). Already 30 min after injection of 10^7^/μl PWL significantly dropped from 13.62 ± 0.66 s at baseline to 3.31 ± 0.22 s (*p* < 0.001; Mann-Whitney *U*-test). Injection of 10^5^/μl induced a maximal reduction in PWL of 18.78 ± 5.88%, 10^6^/μl led to a maximal reduction in PWL of 31.01 ± 6.76% and 10^7^/μl resulted in a maximal reduction of 75.26 ± 2.24%. Quantification of PWL 1 h after injection showed significant differences between the different concentrations with a positive linear correlation (*r*^2^ = 0.9845, Figure 1C). The strongest effect was seen after injection of platelets with a concentration of 10^7^/μl. Over the course of several hours after the injection, PWL recovered slowly and reached baseline values after 24 h.

Since platelets release their content after activation and it was anticipated that one or more of the factors released were responsible for the proalgesic action, we assessed whether the supernatant of platelets activated by sonication likewise affected heat sensitivity *in vivo*. Similar to activated platelets, the injection of the supernatant induced a significant reduction in PWL from 13.13 ± 0.55 s at baseline to 6.24 ± 0.19 s within 30 min after injection (*p* < 0.05; Mann-Whitney *U*-test, Figure 1B). The time course of heat hypersensitivity in response to injection of the supernatant was similar to the platelet preparations and the degree of hypersensitivity was greater than the change observed with 10^6^/μl but less severe than for the highest concentration (10^7^).

Like the majority of cells and tissues, circulating platelets generate S1P as the final product of the tightly regulated ceramide to S1P pathway (Maceyka et al., 2002). Compelling evidence implicates this pathway as a contributor to pain of diverse etiologies (Mair et al., 2011; Camprubí-Robles et al., 2013; Salvemini et al., 2013). Circulating platelets are a major source of extracellular S1P and concentrations of 0.2–0.9 μM (75–340 ng/ml) and 0.4–1.1 μM (150–415 ng/ml) have been reported for blood plasma and serum, respectively, although S1P is largely bound to albumin and high-density lipoproteins (HDL) in these compartments (Caligan et al., 2000; Murata et al., 2000; Berdyshev et al., 2005; Spiegel and Milstien, 2011). We therefore quantified the S1P content of different platelet preparations with LC-MS/MS. As shown in Figure 1D, S1P content significantly correlated with platelet numbers (*r*^2^ = 0.999; 10^5^/μl: 1.85 ± 0.65 ng/ml, 10^6^/μl: 14.54 ± 2.73 ng/ml, 10^7^/μl: 130.15 ± 35.38 ng/ml). In addition, the supernatant of sonicated platelets contained 23.11 ± 2.57 ng/ml S1P, suggesting that S1P was released from platelets upon activation in physiologically relevant concentrations.

### Role of S1P receptors 1 and 3 in mediating heat hypersensitivity after injection of activated platelets

Of the five G protein coupled receptors of the S1P family known to date (Chun et al., 2010), three have been located in primary sensory neurons. While S1P_1_ is largely restricted to small diameter neurons giving rise to nociceptors, S1P_2_ is present predominantly in large DRG neurons giving rise to low threshold mechanosensors, which are insensitive to thermal stimuli. The S1P_3_ receptor, in contrast, is expressed in virtually all sensory neurons including peptidergic and non-peptidergic nociceptors (Camprubí-Robles et al., 2013).

**Figure 2:**
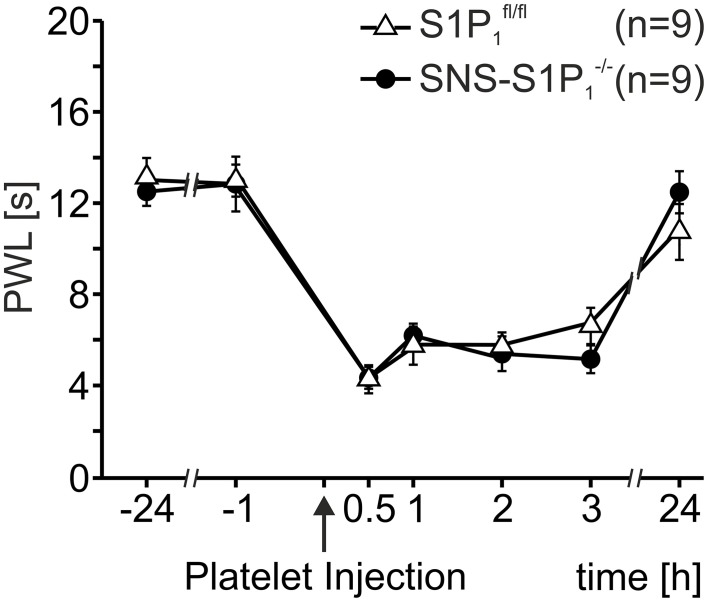
**Thermal hypersensitivity after injection of activated platelets not mediated through the S1P_1_ receptor**. After intracutaneous injection of platelet preparations (10 μl; 10^7^ platelets/μl) no differences in PWL reduction were detected between SNS-S1P_1_^−/−^ and S1P^fl/fl^ control mice (Two-Way Repeated Measures ANOVA: n.s.).

Therefore, we assessed platelet induced heat hypersensitivity in SNS-S1P_1_^−/−^ mice, which lack the S1P_1_ receptor specifically in Na_v_1.8 expressing primary nociceptive afferents. As shown in Figure 2, injection of activated platelets (10^7^/μl) led to similar reactions in both SNS-S1P_1_^−/−^ and S1P_1_^fl/fl^ control mice (Two-Way Repeated Measures ANOVA: n.s.) suggesting that S1P_1_ receptors expressed by nociceptors do not contribute to the platelet induced heat hypersensitivity.

**Figure 3:**
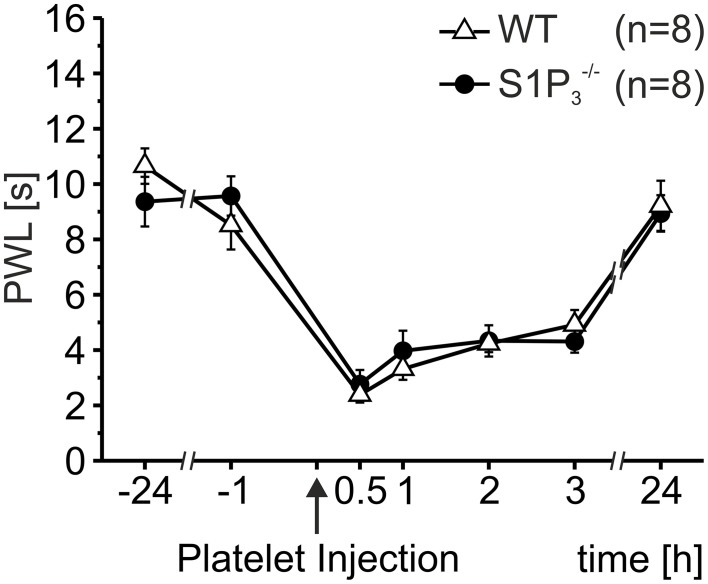
**Thermal hypersensitivity after injection of activated platelets not mediated through the S1P_3_ receptor**. After intracutaneous injection of platelet preparations (10 μl; 10^7^ platelets/μl) no differences in PWL reduction were obtained between S1P_3_^−/−^ and wild-type (WT) control mice (Two-Way Repeated Measures ANOVA: n.s.).

In addition to the S1P_1_ receptor, small sensory neurons express the S1P_3_ receptor and it has been shown that these receptors mediate ongoing pain behavior induced by S1P injections in mice and humans (Camprubí-Robles et al., 2013). However, activated platelets (10^7^/μl) induced significant hypersensitivity to heat stimulation in both S1P_3_^−/−^ and wild-type control mice, and the degree of hypersensitivity was similar for S1P_3_^−/−^ and wild-type control mice (Two-Way Repeated Measures ANOVA: n.s.; Figure 3). This suggests that S1P released by activated platelets does not directly affect nociceptors to induce nociceptor sensitization and hypersensitivity to heat.

### Role of S1P as mediator of platelet induced heat hypersensitivity

**Figure 4:**
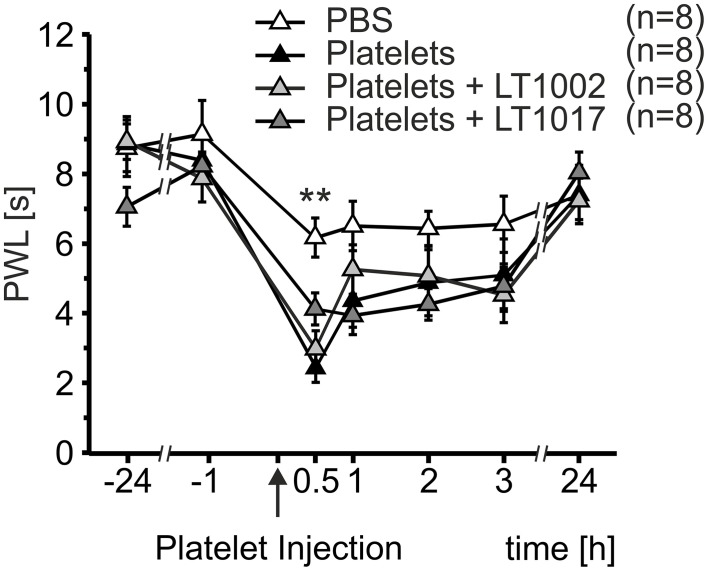
**Thermal hypersensitivity after injection of activated platelets not mediated through indirect mechanisms of S1P**. Intracutaneous injection of platelet preparations (10 μl; 107 platelets/μl) with or without a neutralizing anti-S1P antibody (LT1002) or an inactive antibody (LT1017) led to a strong reduction in PWL, whereas injection of PBS resulted in a minor reduction in PWL (Two-Way Repeated Measures ANOVA with Bonferroni *post-hoc* test, ^**^p < 0.01 vs. PBS).

Since heat hypersensitivity was preserved in both transgenic mouse models, a possible indirect mechanism of nociceptor sensitization was anticipated for S1P released from platelets. In order to test this hypothesis, platelet preparations were mixed with either the murine monoclonal anti-S1P antibody, Sphingomab/LT1002, or its isotype mAB control, LT1017, prior to injection. Necessary antibody concentrations were calculated based on the S1P content of platelet preparations measured by LC-MS/MS. Platelet preparations containing the anti-S1P antibody, Sphingomab/LT1002, as well as preparations containing the control antibody, LT1017, or no antibody, led to a significant decrease in PWL 30 min after injection, when compared to PBS control injections (Figure 4). Injection of PBS induced a maximal reduction in PWL of 30.06 ± 7.06%, platelets led to a maximal reduction in PWL of 70.35 ± 4.12%, platelets + LT1002 resulted in a maximal reduction in PWL of 61.92 ± 6.00% and platelets + LT1017 brought about a maximal reduction in PWL of 49.02 ± 6.04% (Two-Way Repeated Measures ANOVA with Bonferroni *post-hoc* test, ^**^p < 0.01 vs. PBS). This suggests, that S1P released by activated platelets did not play a major role in platelet-induced heat hypersensitivity.

## Discussion

In this study we provide evidence that activated platelets had a pronociceptive effect *in vivo.* We demonstrate that mice injected with platelet preparations of increasing concentrations displayed an augmented sensitivity to heat stimuli in a dose-dependent manner, which was detectable for several hours after injection. Despite the substantial amounts of S1P that were released by platelets upon activation, the platelet induced heat sensitization was independent not only of S1P_1_ and S1P_3_ receptors expressed in nociceptors, but more importantly, S1P did obviously not contribute to platelet induced nociceptor sensitization.

Earlier studies have demonstrated that platelets are capable of driving a subset of primary afferent neurons that sense potentially noxious events, the nociceptors. For example, activated human platelets excite mechano-heat responsive C-nociceptors in an *in vitro* skin-nerve preparation of rat hairy skin (Ringkamp et al., 1994). Furthermore, intracutaneous injections of activated platelets resulted in acute pain and hyperalgesia in human volunteers (Schmelz et al., 1997; Blunk et al., 1999).

Although platelets are known to play essential roles during inflammatory processes and have been shown to contain a plethora of pro-inflammatory and anti-inflammatory cytokines and chemokines, proteases, ADP, ATP, histamine, 5-HT, TXA2, PAF, IL-1β, and S1P (reviewed in Horstman et al., 2010; Semple et al., 2011; Ware et al., 2013), the nature of the mediators that are most relevant for nociception have yet to be elucidated. Several of these pro-inflammatory mediators have been assessed in various models of pathological pain conditions including inflammatory pain (Basbaum et al., 2009). ATP has been extensively studied in the context of nociceptor activation and sensitization (reviewed in Bland-Ward and Humphrey, 2000; Burnstock, 2000; Wirkner et al., 2007) and may also directly or indirectly modulate TRPV1 (Lishko et al., 2007).

Some mediators released from platelets have been shown to act synergistically with other factors, such as bradykinin (BK) (Koppert et al., 1993) and TXA2 (Hori et al., 1986). The BK receptors may be upregulated upon tissue injury or after the release of cytokines such as IL-1β (Marceau et al., 1998), which is also stored in and released from platelets. Another classical algogen which sensitizes nociceptors is 5-HT (Lang et al., 1990; Taiwo and Levine, 1992; Babenko et al., 2000; Ernberg et al., 2000, 2006; Schmelz et al., 2003). Finally, platelet activating factor (PAF) induces spontaneous nociception and mechanical hypersensitivity in addition to its known functions during inflammation and hemostasis (Teather et al., 2002; Marotta et al., 2009).

In addition to these mediators, circulating platelets together with red blood cells are generally accepted as a major source of extracellular S1P in blood (Yatomi, 2008). In the present study, platelet preparations contained substantial amounts of this bioactive sphingolipid and S1P was released into the supernatant after activation. So far the S1P release mechanism from platelets has not fully been elucidated, but platelets contain two releasable pools of S1P: a non-granular one for the constitutive release without stimulation and a granular one that is released upon activation and requires regulated exocytosis (Jonnalagadda et al., 2014). In addition, platelets are known to express the ATP-binding cassette transporter 4 (ABCC4) protein (Jedlitschky et al., 2004) and the release of S1P from human platelets can be blocked with the ABCC transporter-inhibitor MK751 (Ulrych et al., 2011). In line with these reports, S1P was detectable in physiologically relevant concentrations after platelet activation by freeze/thawing *in vitro*, which has been established to be a very effective method of activation (Rauch et al., 2011).

However, it remains elusive whether similar concentrations of S1P at the nociceptors are reached upon platelet injection into the mouse hind paw, but it can be anticipated that 80% of the S1P contained within the platelets is released within 5 min after activation as it has been shown in *in vitro* studies (Kobayashi et al., 2006; Jonnalagadda et al., 2014) We hypothesized that the pronociceptive effect of activated platelets *in vivo* was based on S1P release and downstream activation of S1P receptors located in the plasma membrane of nociceptive neurons. This paracrine signaling of S1P is important in inflammatory processes where free S1P levels increase substantially at the site of inflammation (Spiegel and Milstien, 2003; Takabe et al., 2008).

In dorsal root ganglia (DRG), where the cell bodies of nociceptive neurons are located, all five S1P receptors are expressed (Kays et al., 2012), but small diameter nociceptive neurons mainly express S1P_1_ and S1P_3_ (Mair et al., 2011; Camprubí-Robles et al., 2013). Several lines of evidence support the idea that increased heat hypersensitivity after S1P injection is mediated by the S1P_1_ receptor located on primary nociceptive afferents (Doyle et al., 2011; Mair et al., 2011; Xie et al., 2012). After injection of S1P into the hindpaw the resulting heat hypersensitivity is significantly reduced in SNS-S1P_1_^−/−^ mice as compared to S1P_1_^fl/fl^ control mice. At the same time, S1P_3_ activation induces nociceptor excitation with action potential firing in a cellular pain model and spontaneous pain behavior *in vivo* and S1P_3_^−/−^ display a significantly reduced spontaneous pain behavior and recover significantly faster from post-surgical pain (Camprubí-Robles et al., 2013).

Surprisingly, the heat hypersensitivity occurring after platelet injection was fully preserved in both knock-out mouse strains in the current study. Despite converging evidence for a proalgesic and receptor-mediated action of S1P, S1P receptors expressed in nociceptive neurons were not critically involved in the pronociceptive action of platelets reported here. This could have been explained by spatio-temporal confinement, which has become a generally accepted mechanism for the regulation of complex biochemical networks (Shen et al., 2009). In particular tight binding of S1P to the components of serum or plasma, including lipoproteins, may interfere with S1P binding to its receptors and thereby attenuate the lipid-receptor-mediated actions in the cells (Murata et al., 2000). The strong association of platelets with apolipoproteins and other components of HDL, which also behave as carriers for S1P, together with the HDL complex seems to have effects that are completely different from those observed for free S1P and this is currently extensively studied in the cardiovascular system (Scanu and Edelstein, 2008; Kennedy et al., 2009). However, the lack of effect of S1P neutralization rather suggests that S1P released from activated platelets does not play a role in nociceptor sensitization.

## Conclusions

In the present study we provide evidence that activated platelets have a pronociceptive effect and thus may be implicated in post-operative pain. Although platelets contain and release substantial levels of S1P, an action of S1P, either directly via neuronally expressed receptors S1P_1_ and S1P_3_ or via indirect mechanisms, for the establishment of the observed heat hypersensitivity of nociceptors was not supported in the current settings. Although S1P and its receptors S1P_1_ and S1P_3_ are critically important regulators of nociception for which pronociceptive effects via direct regulation of nociceptors have been reported previously (Mair et al., 2011; Camprubí-Robles et al., 2013) the pronociceptive effect of activated platelets involves mediators other than S1P to modulate nociceptor heat sensitivity.

## Author contributions

Conceived and designed the experiments: DW, CB, GGs, GGe, RS, MK. Performed the experiments: DW, CB, HS. Analyzed the experiments: DW, CB, HS, GGe, MK. Contributed reagents/materials/analytic tools: CR, RP, GGs, GGe, RS, MK. Wrote the manuscript: DW, RP, GGs, GGe, MK. All authors read and approved the final manuscript.

### Conflict of interest statement

The authors declare that the research was conducted in the absence of any commercial or financial relationships that could be construed as a potential conflict of interest.
